# Skin Lesion Classification Using Additional Patient Information

**DOI:** 10.1155/2021/6673852

**Published:** 2021-04-10

**Authors:** Qilin Sun, Chao Huang, Minjie Chen, Hui Xu, Yali Yang

**Affiliations:** ^1^Department of Dermatology, Shanghai Ninth Hospital affiliated to Shanghai Jiao Tong University, School of Medicine, No. 639, Manufacturing Bureau Road, Huangpu District, Shanghai 200011, China; ^2^Department of Orthopaedics, West China Hospital of Sichuan University, No. 37 Guoxue Alley, Wuhou District, Chengdu, 610041 Sichuan, China; ^3^Zeku Technology Co., Ltd., 8th Floor, Building 1, No. 61, Shen Xia Road, Pudong New District Shanghai 201203, China

## Abstract

In this paper, we describe our method for skin lesion classification. The goal is to classify skin lesions based on dermoscopic images to several diagnoses' classes presented in the HAM (Human Against Machine) dataset: melanoma (MEL), melanocytic nevus (NV), basal cell carcinoma (BCC), actinic keratosis (AK), benign keratosis (BKL), dermatofibroma (DF), and vascular lesion (VASC). We propose a simplified solution which has a better accuracy than previous methods, but only predicted on a single model that is practical for a real-world scenario. Our results show that using a network with additional metadata as input achieves a better classification performance. This metadata includes both the patient information and the extra information during the data augmentation process. On the international skin imaging collaboration (ISIC) 2018 skin lesion classification challenge test set, our algorithm yields a balanced multiclass accuracy of 88.7% on a single model and 89.5% for the embedding solution, which makes it the currently first ranked algorithm on the live leaderboard. To improve the inference accuracy. Test time augmentation (TTA) is applied. We also demonstrate how Grad-CAM is applied in TTA. Therefore, TTA and Grad-CAM can be integrated in heat map generation, which can be very helpful to assist the clinician for diagnosis.

## 1. Introduction

Skin cancer is the most common cancer around the world. Early detection and monitoring play a crucial role decreasing the mortality rate of skin cancer. However, it is a challenging problem that only 65%-80% of the skin cancer cases are correctly diagnosed using clinical inspection by an experienced physician [1]. Perez et al. investigated the impact of 13 data augmentation scenarios, such as traditional color and geometric transforms, elastic transforms, random erasing, and lesion mixing method for melanoma classification. The results confirmed that data augmentation can lead to more performance gains than obtaining new images. Recently, a challenge from the international skin imaging collaboration (ISIC) Skin Lesion Analysis Towards Melanoma Detection resulted in numerous high-performing methods that performed similar to human experts for the evaluation of dermoscopic images, which was mostly based on the convolutional neural network technique [2]. And most of those methods obtain a high classification accuracy through ensembling multiple models [3, 4]. For example, 18 convolutional neural network (CNN) architectures [4] and 7 multiresolution EfficientNet (B4) models [3] are explored, with extensive data augmentation. The final results are obtained by 90 submodels, which takes 13.9 seconds to classify single test images for high-end TitanV graphic card [5]. However, in these ensembling method, every image has to be sent through all the models for inference process, and this scheme will even run multiple times on each model for data augmentation, which have a significant amount of computation and would not be practical for a real-world scenario.

Here, we propose a simplified solution which has a better accuracy than previous methods and is also practical for a real-world scenario.

## 2. Method Details

### 2.1. Datasets

The ISIC 2019 skin lesion classification challenge dataset contains 25, 331 dermoscopic images, with extra meta information about the patients' age, the anatomical site, and the sex properties [6–8]. We also use 1,572 extra images in training, including 170 images from the MED-NODE dataset, 533 from the seven-point dataset, 120 from the PH2 dataset, and the remaining ones are our own collected data.

### 2.2. Image Preprocessing

We resize all images' longer side to 1,024 pixels while preserving the aspect ratio. The shades of gray color constancy method proposed by Finlayson and Trezzi is applied beforehand as a preprocess step, and its color gain in RGB channel is recorded as extra metainfo input. [3]

### 2.3. Additional Patient Information Preprocessing

The patient's additional patient information is mostly encoded by a one-hot encoding scheme. For example, six features are used for the anatomical site with enough appearance (>100). Sex is encoded as 1/-1/0 for man/female/missing, respectively. Age is encoded by 18 features, under 18 thresholds from 5-90 with step 5, where 1/-1/0 represented larger/smaller/missing, respectively.

### 2.4. CNN Architectures

**Figure 1:**
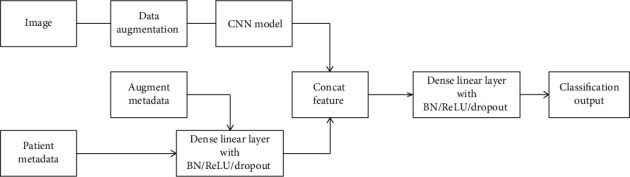
Architecture of the proposed CNN model with metadata.

We use EfficientNet that have been pretrained on the ImageNet dataset [9]. This model family contains 8 different models that are structurally similar and follow certain scaling rules for adjustment to larger image sizes, from the smallest version B0 to larger versions, up to B7 **(**Figure 1**)**. To incorporate additional patient information such as age, anatomical site, and sex, an additional dense neural network and fuse its features with the CNN is discussed [4]. In our experiment, we reported the performance of a single model B4, as well as an ensemble model with B3 and B4. We use the default input size described in EfficientNet paper, which is 300 × 300 for B3 and 380 × 380 for B4.

### 2.5. CNN Data Augmentation

We perform data augmentation in training, both geometric and pixelwise, including random brightness, contrast, hue, saturation, Gaussian Noise, Gaussian blur, random crop, rotation, and flipping. Moreover, we also recorded the scaling and shifting properties of the geometric augmentation which was later used as the metafeatures during the training process. These augmentation processes were implemented for using the albumentations.

### 2.6. CNN Training

We train the models for 60 epochs with batch size 16 using SGD with momentum. One cycle learning-rate scheduler was applied [10], which was implemented by pytorch OneCycleLR function with default parameter, and the learning rate was set as 1*e* − 3. A weighted cross-entropy loss function where underrepresented classes receive a higher weight. The coefficient was calculated by the formula described by Lin et al. [11] Focal loss with a gamma = 1.5 was also tested; unfortunately, the testing accuracy was lower [12]. Training was performed on NVIDIA GTX 1080TI. A sampling strategy was applied during the dataloader procedure. In the metafile, a lesion ID was provided for each image. For one lesion, there were 1-30 images in the dataset and those images for the same lesion ID were with high similarity. Therefore, a sampling weight coefficient was added which was equal to inverse of the number of images for that lesion ID.

## 3. Discussion

Skin cancer is one of the most common malignancy with an increasing incidence rates on a global scale. [13] Early detection is an important factor to increase the overall survival and cure rates for those patients [5]. The diagnosis of those diseases is usually carried out by dermatologists through the visual examination of suspicious skin areas, but it is easy to be misdiagnosed due to the high similarities of some types of lesions. Although supportive imaging techniques such as dermoscopy can improve the accuracy of diagnosis to some extent, the accuracy of diagnosis varies greatly among individuals with different experience.

A large number of studies have been devoted to improving the accuracy of diagnosis and treatment. In recent years, more and more semiautomatic or fully automatic computer-aided diagnosis (CAD) systems based on classical image processing techniques or advanced machine learning paradigms, such as classical workflow of machine learning and CNNs, have been introduced into the diagnosis and treatment of skin diseases as screening procedures or rapid diagnosis tools to assist dermatologists [1, 4, 14]. In addition, the quality of classification could be improved by adding clinical data (such as age, sex, race, skin type and anatomical location) as input to the classifier, and this additional information is helpful for dermatologists to make the right decisions [15]. Perez et al. confirmed that data augmentation can lead to more performance gains than obtaining new images, which was based on the researches of the impact of 13 data augmentation scenarios, such as traditional color and geometric transforms, elastic transforms, random erasing, and lesion mixing method for melanoma classification [16]. Although these methods improve the accuracy of diagnosis and treatment, they all have a significant amount of computation and the application value in practical operation still needs to be improved.

**Figure 2:**
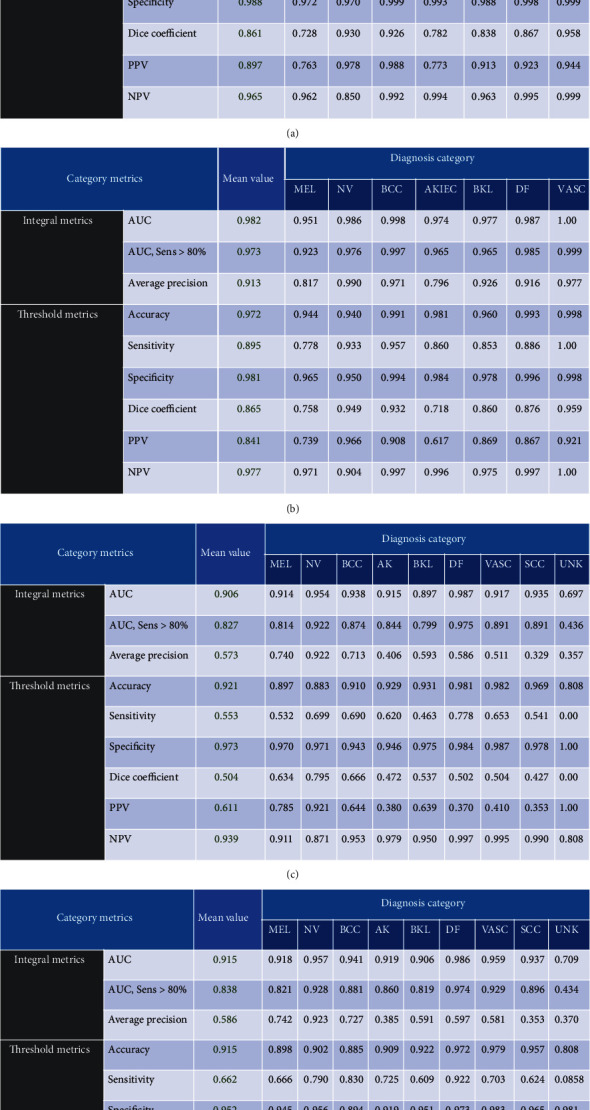
The metrics of all lesion type reported on ISIC live leaderboard: (a) our single model on ISIC18, (b) our embedding model on ISIC18, (c) our single model on ISIC19, and (d) our embedding model on ISIC19.

In this study, we propose a simplified solution and have evaluated our proposed method on both ISIC 2018 and ISIC 2019 test set (Figure 2). The final predicted probability is archived by 10 times TTA, which costs approximated 0.5 second on GTX 1080Ti. There is a live leaderboard to record the performance of the submitted result [2]. The balanced multiclass accuracy (BMCA) is used as the primary metric value, which is shown on Tables 1 and 2. Our results show that using a network with additional patient information as an input achieves a better classification performance. On the ISIC 2018 skin lesion classification challenge test set, our algorithm yields a balanced multiclass accuracy of 88.7% on a single model and 89.5% for the embedding solution, which makes it the currently first ranked algorithm on the live leaderboard and highlights the excellent performance of our proposed solution on this very challenging task (Table 1). Similarly, we also saw the superiority of our proposed method on the ISIC 2019 test set (Table 2). We also observed that weighted cross-entropy criterion achieves higher BMCA than focal loss or label smooth loss criterion.

Grad-CAM is an efficient method to generate the heat map for visualizing where is the hot zone for the classification, which is quite helpful for assisting the clinicians during the diagnosis [17]. To improve the inference accuracy, test time augmentation (TTA) is also applied.

In this paper, we proposed an integrated solution for Grad-CAM and TTA with multicrop. This is implemented by accumulating the heat map on multiple inference process during TTA. As random crop is included in TTA, the final heat map is accumulated for different crop regions with different resolution. As TTA is an efficient way to improve the final prediction metrics; therefore, we believed the weighted heat map generated by this TTA-Grad CAMs operation will also have benefits for the diagnosis of the clinicians.

**Figure 3:**
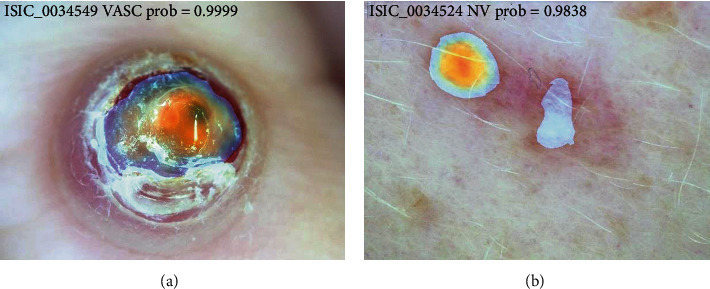
Heat map visualization using Grad-CAM with TTA. The figures are from the ISIC competition (https://challenge2019.isic-archive.com/data.html).

An example can be seen in Figure 3. The color is the heat map generated by applying Grad-CAM with TTA using the training model. The redder it is, the more likely that the area is a type of disease diagnosed by a neural network. Therefore, TTA and Grad-CAM can be integrated in heat map generation, which can be very helpful to assist the clinician for diagnosis.

We also test the semisupervised scheme as described by utilizing additional unlabeled testing images in the training process. [18] However, we found that the overall accuracy had not been improved. We also tested other advanced augmentation methods, such as Cutmix [19], as well as some attention-based method WS-DAN [20], which achieved the best result in fine-grained image classification tasks, but the performance has not improved.

## 4. Conclusion

In this study, we have proposed a single baseline for skin lesion classification which uses the information of data augmentation as additional patient information. The metadata used in our manuscript included additional infos that are generated during data augmentation, for example, gain of color normalization process, random crop, and image size properties. Our method has achieved the best result of ISIC live leaderboard with a balanced multiclass accuracy of 88.7% on a single model and 89.5% for the embedding solution, making it the currently first ranked algorithm on the live leaderboard. In addition, it is also practical for real application because of its low computational complexity.

## Figures and Tables

**Table 1:** Results of ISIC 2018 challenge winners from the legacy leaderboard (rows 1-3) and our proposed models (rows 4–6). Among the 16,888 images, there are 15,316 images from ISIC19 dataset, 170 images from the MED-NODE dataset, 533 from the seven-point dataset, 120 from the PH2 dataset, and the remaining data are from our own collected data.

Team/authors	Extra images	BMCA (%)	Sensitivity (%)	Specificity (%)	AUC
Nozdryn et al.	37,807	88.5	83.3	98.6	0.983
Gassert et al. [14]	13,475	85.6	80.9	98.4	0.987
MSM-CNN [5]	2,912	86.2	85.6	97.9	0.987
Our single model (FL)	16,888	88.3	76.1	99.3	0.974
Our single model (CE)	16,888	*88*.*7*	*83*.*0*	*98*.*8*	*0*.*981*
Our ensemble model	16,888	*89*.*5*	*89*.*5*	*98*.*1*	*0*.*982*

FL: focal loss; CE: cross-entropy loss.

**Table 2:** Results of ISIC 2019 challenge winners from the legacy leaderboard (rows 1-3) and our proposed models (rows 4–6). Among the 1,572 images, there are 170 images from the MED-NODE dataset, 533 from the seven-point dataset, 120 from the PH2 dataset, and the remained data are from our own collected data.

Team/authors	Extra images	BMCA (%)	Sensitivity (%)	Specificity (%)	AUC
Gassert et al. [14]	Unknown	63.6	50.7	97.7	0.923
Cancerless	Unknown	63.8	53.1	97.4	0.913
ForCure	Unknown	64.8	53.4	97.4	0.914
Our single model (FL)	1,572	63.9	48.8	97.9	0.899
Our single model (CE)	1,572	*65*.*0*	*55*.*3*	*97*.*3*	*0*.*906*
Our ensemble model	1,572	*66*.*2*	*66*.*2*	*95*.*2*	*0*.*915*

FL: focal loss; CE: cross-entropy loss.

## Data Availability

The data used to support the findings of this study are included within the article.
